# Denser Retinal Microvascular Network Is Inversely Associated With Behavioral Outcomes and Sustained Attention in Children

**DOI:** 10.3389/fneur.2021.547033

**Published:** 2021-01-29

**Authors:** Eline B. Provost, Tim S. Nawrot, Luc Int Panis, Arnout Standaert, Nelly D. Saenen, Patrick De Boever

**Affiliations:** ^1^Centre for Environmental Sciences, Hasselt University, Diepenbeek, Belgium; ^2^Health Unit, Flemish Institute for Technological Research (VITO), Mol, Belgium; ^3^Department of Public Health and Primary Care, Leuven University, Leuven, Belgium; ^4^School for Mobility, Hasselt University, Diepenbeek, Belgium; ^5^Department of Biology, University of Antwerp, Antwerp, Belgium

**Keywords:** retina, microvasculature, geometry, fractal dimension, behavior, cognition, children

## Abstract

Changes in geometry of the retinal microvascular network, including vessel width, vessel density, and tortuosity, have been associated with neurological disorders in adults. We investigated metrics of the retinal microvasculature in association with behavior and cognition in 8- to 12-year-old children. Digital fundus images of 190 children (48.2% girls, mean age 9.9 years) were used to calculate retinal vessel diameters, fractal dimension, lacunarity, and tortuosity. Parents filled out a Strengths and Difficulties Questionnaire (SDQ) for behavioral screening. Cognitive performance testing included a computerized version of the Stroop test (selective attention), the Continuous Performance (sustained attention), the Digit-Symbol (visual scanning and information-processing speed) and the Pattern Comparison (visuospatial analytic ability) tests from the Neurobehavioral Evaluation System (NES3) battery. Retinal vessel geometry was significantly associated with the SDQ problem score, which increased with 1.1 points (95% CI: 0.3 to 1.9 points) per interquartile (IQR) increment in retinal fractal dimension, and decreased 1.4 points (95% CI: −2.4 to −0.4 points) or decreased 1.0 points (95% CI: −2.1 to 0.1 points) per IQR increment in retinal vascular lacunarity or tortuosity, respectively. Sensitivity analyses showed that results were driven by the hyperactivity/inattention and conduct problem scales of the SDQ. Correspondingly, mean reaction time on the Continuous Performance test increased by 11 ms (95% CI: 4.4 to 17.6 ms) with an IQR increase in fractal dimension. The results indicate that a denser retinal microvascular network, exemplified by a higher fractal dimension and lower lacunarity, are inversely associated with behavioral outcomes and sustained attention in children.

## Introduction

Retinal vessel morphology changes are linked to cerebrovascular and neurobehavioral disorders (1–6). Indeed, the retinal and cerebral microcirculation have similarities with respect to morphology and physiology (7), with concomitant changes present in both microvascular beds of patients (8, 9). In this context, the status of the retinal microvasculature can be quantified with several retinal vessel metrics that can be calculated from a digital fundus image. Retinal vessel width is a frequently used metric, but retinal vessel geometric properties such as vessel branching complexity, vessel density, as assessed by fractal dimensions, and tortuosity are gaining importance (10, 11).

Retinal vessel analysis has been introduced into clinical research in adults in the field of different neurological disorders such as schizophrenia, stroke, dementia, and Alzheimer's disease (12–19). The few studies with young children suggest that retinal microvascular changes can be related to behavioral characteristics or cognitive performance. Compared to an age- and sex-matched reference group, children with attention deficit hyperactivity disorder (ADHD) showed a decreased tortuosity of their retinal arteries (20). Results from a longitudinal cohort study showed wider retinal venular diameters in association with both poorer neuropsychological functioning at midlife and a lower childhood IQ tested 25 years earlier (21). In addition, wider retinal venular diameters were also associated with psychosis symptoms in a longitudinal twin-study (22). A decrease in total IQ, matrix reasoning and spatial span was associated with smaller retinal arteriolar vessel diameters in 11-year old children (23). A recent study performed in children aged 4–5 years found retinal venular widening and a higher vessel tortuosity in association with a lower performance of short-term visual recognition memory (24). In the current study, we investigated whether properties of the retinal microvascular network, including retinal vessel width and geometric complexity, could be associated with behavior and cognitive performance outcomes in a panel of primary school children.

## Materials and Methods

### Study Population

This research was part of the COGNAC (COGNition and Air pollution in Children) study (25). Children aged 8–12 years from two primary schools in Flanders (Belgium) were invited for repeated clinical examinations conducted at their school. The two schools were 3.7 km apart and located in the agglomeration of Hasselt (~70 km east from Brussels). Of the 482 invited children, 221 (46%) agreed to participate of which 72 (33%) underwent three clinical examinations, 124 (56%) completed two examinations and 25 (11%) had only one examination, amounting to a total of 489 examinations. The on-site examinations were done during school years 2012-2013 for one school and 2013-2014 for the other, from November to February on Monday, Tuesday, Thursday, and Friday between 8:30 a.m. and 3:30 p.m. The average (SD) time between two consecutive examinations was 49 (19) days. The clinical examinations of each child were scheduled on the same time of the day and day of the week to minimize circadian variation. Clinical examinations were performed by a trained examiner and included assessment of cognitive performance, acquisition of fundus images, and measurement of blood pressure and heart rate.

We conducted the study according to the principles outlined in the Helsinki declaration for research on human participants. The ethics committees of Hasselt University and Ziekenhuis Oost-Limburg approved the study. Written informed consent was obtained from the parents as well as oral assent from the children. The parents filled out a questionnaire addressing aspects related to sociodemographic and medical characteristics of the child and its family.

Additional information on the indoor and outdoor environment of the residence, including current smoking status of the parents, was collected.

### Behavior Assessment

Parents filled out a Strengths and Difficulties Questionnaire (SDQ). The SDQ is a 25-item behavioral screening questionnaire for children between 3 and 16 years old. The 25 items are divided between five scales (5 items each): emotional symptoms, conduct problems, hyperactivity/inattention, peer relationship problems, and prosocial behavior. The first four scales combined generate a total difficulties score (based on 20 items). Research has shown that increases in this overall score increase the odds for clinical mental disorders in children, such as autism spectrum disorder (ASD) and attention deficit hyperactivity disorder (ADHD) (26, 27).

### Cognitive Performance Assessment

**Figure 1:**
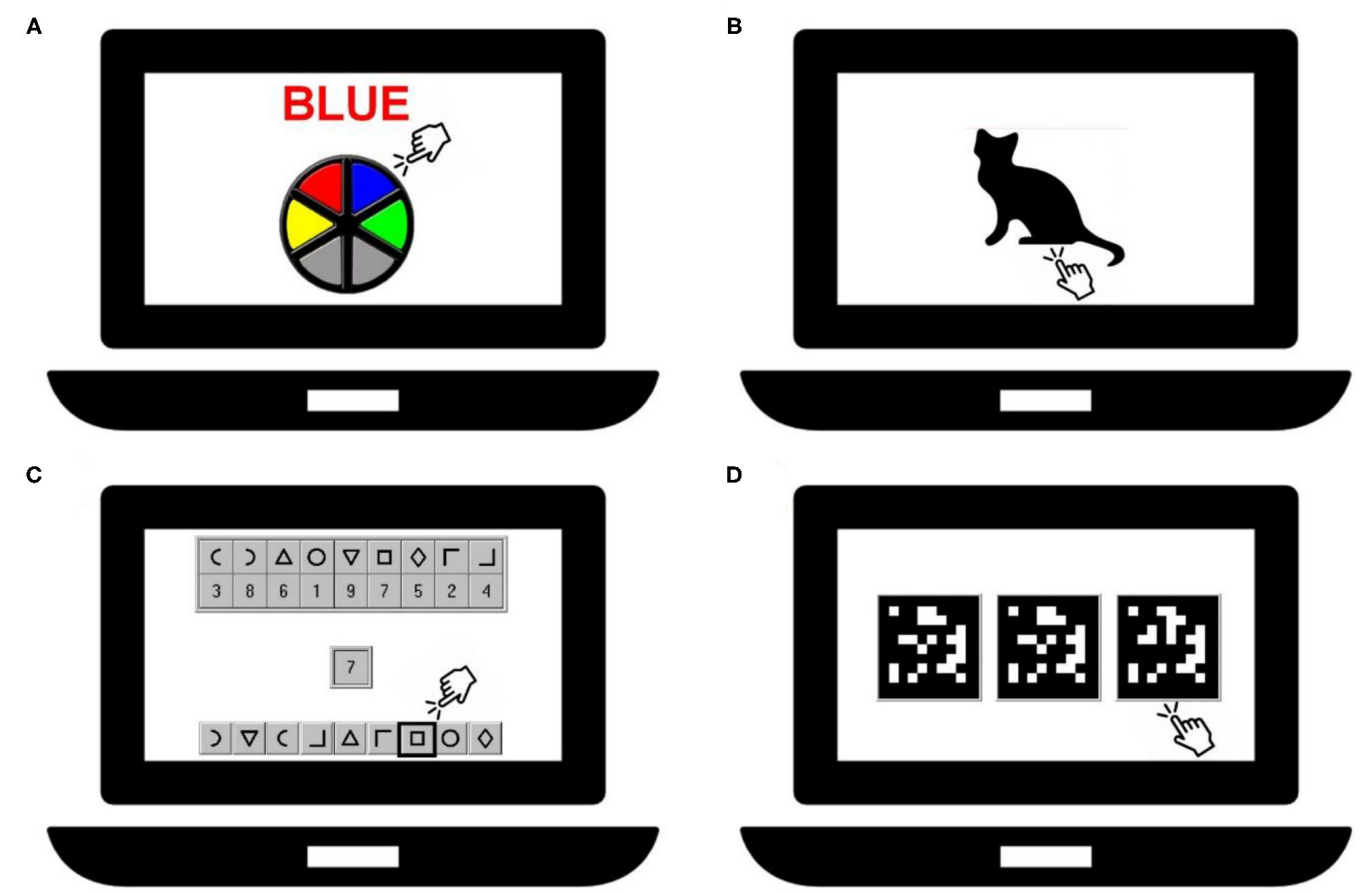
Representation of the cognitive tests: Stroop-test **(A)**, Continuous Performance test **(B)**, Digit-Symbol test **(C)** and Pattern Comparison test **(D)**.

Cognitive performance tests were administered in groups of 4 children using an individual touch screen laptop with additional hardware (headset/keyboard). The room in which the examinations took place was quiet, appropriately lighted and ventilated. The duration of the tests was ~20 min. The tests consisted of a computerized version of the Stroop test and the following tests of the Neurobehavioral Evaluation System (NES3) battery: Continuous Performance, Digit-Symbol Substitution, and Pattern Comparison test. A depiction of the tests is given in Figure 1 and they are also described by Saenen et al. (25). The Stroop test (Dutch translated version from Xavier Educational Software Ltd, UK) assesses selective attention by presenting the name of colors in a color not denoted by the name. During the test, the name of a color appears on the screen printed in a different color than the name. The task is to touch as fast as possible the button that has the same color as the name, ignoring the color of the printed name. The primary summary measure was the mean reaction time in milliseconds between the appearance of the name and touching the correct button. The children were presented 48 trials.

The Continuous Performance test measures sustained attention. Silhouettes of animals (e.g., a cat) are displayed on the screen, one at the time and each for ~200 ms. The task is to immediately respond to the cat's silhouette in this case by pressing the spacebar, but not the silhouette of another animal. A new silhouette is displayed each 1,000 ms. The primary summary measure was the mean reaction time in milliseconds for responding to the correct silhouette. Thirty-six trials were presented to the children.

The Digit-Symbol Substitution test assesses visual scanning and information-processing speed. A row of 9 symbols paired with 9 digits is shown at the top of the screen. The same 9 symbols but in a different order are displayed at the bottom of the screen. During the test 27 digits appear consecutively on the screen. When a digit is shown, the task is to indicate as fast as possible the symbol which is paired with this digit in the row of symbols at the bottom of the screen. A new digit appears only after the correct symbol has been indicated. The primary summary measure was the latency in seconds to complete responses to 27 target digits.

The Pattern Comparison test assesses visuospatial analytic ability. Three matrices consisting of 10 by 10 blocks were presented of which two were identical. The task was to indicate which of the patterns was different from the other two. The primary summary measure was the average response latency in seconds of the items answered correctly. A test included 25 items.

### Retinal Imaging and Analysis

The fundus of the left and right eye of each participant were photographed with a Canon 45° 6.3-megapixel digital non-mydriatic retinal camera (Hospithera, Brussels, Belgium) as described by De Boever et al. (28). The widths of the retinal arterioles and venules that pass completely through the circumferential zone 0.5–1 disc diameter from the optic disc margin were calculated automatically from the digital fundus images using IVAN software. A trained grader verified and corrected vessel diameters and vessel labels (arteriole or venule) with the IVAN vessel editing toolbox. The diameters of the 6 largest arterioles and 6 largest venules were used in the revised Parr-Hubbard formula for calculating the Central Retinal Artery Equivalent (CRAE) and Central Retinal Venular Equivalent (CRVE) according to previously reported protocols (29).

Geometric features of the retinal microvasculature were extracted from the digital fundus images within an area equal to 0.5–2 times the disc diameter from the optic disc margin using vessel analysis software developed at our institute [MONA REVA (version 2.1.1), http://mona.health, VITO (Belgium)]. The MONA REVA algorithm automatically segmented the retinal vessels. The segmentation algorithm is based on a multiscale line filtering algorithm inspired by Nguyen and coworkers (30). Post-processing steps such as double thresholding, blob extraction, removal of small connected regions and filling holes were performed. Complexity of the retinal vessel network was captured from this segmented image by calculating fractal dimension and lacunarity. Fractal dimension represents a direct measure of retinal vessel branching complexity, while lacunarity quantifies how the vessel pattern fills space in the retinal microvascular network.

The monofractal dimension D_f_ was computed using the sliding box-count method (31, 32). Boxes with side length δ slide across the image and for each δ the number of boxes (N) required to cover the segmented image is recorded. The fractal dimension D_f_ is expressed as follows (33):

(1) $$\begin{array}{l} D_{f}=\underset{\delta \to 0}{\lim}-\frac{\log\left(N\right)}{\log\left(\delta \right)} \end{array}$$

Lacunarity is computed like D_f_ but uses the standard deviation of the pixel count for boxes with side length δ that slide across the image. (32).

The tortuosity index, which reflects the curviness of retinal vessels, was computed as the average tortuosity of the branch segments; i.e. the ratio of the line traced on each tree along the vessel axis from 0.5–2 times the disc radius from the optic disc and the line connecting the endpoints. Tortuosity of individual vessel segments was calculated as reported by Lisowska et al. (34). Tortuosity index for the vessel network is similar as in Prabhakar et al. (32).

Intraclass correlation coefficients on the repeated measurements of the geometric properties ranged from 0.72 to 0.88, indicating a good reliability. The microvascular features were averaged over the different examinations to reduce technical variation and data were averaged over both eyes to reduce biological variation within the same child.

### Blood Pressure and Heart Rate Measurement

Blood pressure and heart rate were measured according to the guidelines of the European Society of Hypertension (35). A child rested for 5 min, after which heart rate, systolic (SBP) and diastolic (DBP) blood pressure were measured five times consecutively using an automated upper arm blood pressure monitor (Stabil-O-Graph, EuroMedix, Leuven Belgium) with a special sized cuff for children. The last three measurements were averaged and used to calculate mean arterial pressure.

### Statistical Analysis

SAS software (version 9.4, SAS Institute Inc., Cary, NC, USA) was used for database management and statistical analysis. The association between retinal microvascular features and SDQ problem scores or cognitive performance outcomes was investigated using the PROC GLM procedure.

We adjusted the multiple regression models for an *a priori* chosen list of covariates including sex, age, age-adjusted BMI (36) (categorized as <5th percentile, 5th−85th percentile, 85th−95th percentile, and ≥95th percentile, respectively, corresponding to underweight, normal weight, overweight, and obese), (37) mean arterial pressure, maternal occupation (low: no occupation or blue collar workers; high: white collar workers or self-employed) and passive smoking (yes/no). Both linear and quadratic terms of age were tested. The quadratic term was not significant and was therefore removed from the model. Q-Q plots of the residuals were used to test the assumptions of the model. Of the 221 examined children, 190 (86%) were included in the data analysis. Exclusions were due to underlying medical conditions such as diabetes (*n* = 2) or missing data (*n* = 29); i.e., SDQ questionnaire not filled out, no information on passive smoking or maternal occupation, or low retinal image quality in such that retinal vascular features could not be extracted. Power calculation showed that *n* = 190 was enough to pick up correlations of 0.20, corresponding to 80% power and an alpha of 5%.

Estimates are given as change in SDQ score or mean latency per cognitive performance test associated with an interquartile range (IQR) increment in retinal vessel diameter or fractal dimension, lacunarity, or tortuosity of the retinal microvasculature.

## Results

Details on the study population of 190 school children are summarized in Table 1. There was an approximately equal number of boys and girls. Most of the children had a normal age-adjusted BMI (80.0%), were not exposed to passive smoking (91.6%), and had a high socio-economic status based on the mother's employment (83.2%).

**Table 1:** Description of the study population (*n* = 190).

**Anthropometrics**	
Age	9.9 ± 1.2
Girls	92 (48.2)
Categorized body mass index (BMI)	
Underweight (<5th age-adjusted percentile)	16 (8.4)
Normal weight (5th−85th percentile)	152 (80.0)
Overweight (85th−95th percentile)	17 (9.0)
Obese (≥95th percentile)	5 (2.6)
Mean arterial pressure, mm Hg	80.0 ± 6.5
Exposed to passive smoking	16 (8.4)
Socio-economic status: maternal occupation	
Low (no occupation or blue-collar workers)	32 (16.8)
High (white collar workers or self-employed)	158 (83.2)
Retinal microvasculature metrics	
Fractal dimension	1.500 ± 0.034
Lacunarity	0.495 ± 0.042
Tortuosity	0.857 ± 0.012
Central retinal arteriolar equivalent (CRAE)	163.8 ± 12.2
Central retinal venular equivalent (CVRE)	223.3 ± 16.5

*Values are number (%) or arithmetic mean ± SD. Fractal dimension, lacunarity and tortuosity are given in arbitrary units, Central Retinal Arterial/Venular Equivalent are given in micrometer*.

SDQ problem scores ranged from 0 to 23 with a median (IQR) of 7 (5) points. Most problems were reported in the hyperactivity/inattention scale, with scores ranging from 0 to 10 with a median (IQR) of 3 (4) points.

Mean reaction time (SD) was 1,403 (266) ms for the Stroop test, 598 (48) ms for the Continuous Performance test; Latency (SD) was 126 (22) s for the Digit-Symbol Substitution test and 4.3 (0.9) s for the Pattern Comparison test. The SDQ problem score showed a significant positive correlation with all cognitive performance measures.

Boys had 1.5 points (95% CI: 0.09 to 2.8) higher SDQ problem score than girls. A 1-year increase in age was significantly associated with a decrease in SDQ problem score of 0.8 points (95% CI: 0.2 to 1.4). Children from mothers with a higher level of occupation had 2.2 points (95% CI: 0.3 to 4.1) lower SDQ problem score whereas children passively exposed to tobacco smoke had 2.7 points (95% CI: 0.2 to 5.3) higher SDQ problem score.

A better performance was observed in all cognitive performance tests with increasing age. Occupation of the mother was significantly associated with reaction time in the Stroop test and latency in the Digit-Symbol Substitution test, showing a higher reaction time of 180.1 ms (95% CI: 74.0 to 286.2) and latency of 7.3 s (95% CI: 1.3 to 13.4), respectively, in children from mothers with a lower level of occupation compared to children from mother with a higher level of occupation. Furthermore, girls had a lower latency time of 0.28 s on the Pattern Comparison test compared to boys (95% CI: −0.50 to −0.06).

The study population's average retinal vessel metrics are presented in Table 1. Girls had significantly wider retinal arteriolar and venular diameters compared to boys. A 1 mm Hg higher mean arterial pressure was significantly associated with 0.57 μm narrower retinal arterioles (95% CI: −0.85 to −0.28). Children from mothers with a lower level of occupation had narrower retinal diameters. Age was the only significant determinant of fractal dimension and lacunarity of the retinal microvasculature, showing a decrease in fractal dimension and an increase in lacunarity with increasing age.

Higher mean arterial pressure was associated with 0.003 lower retinal vessel tortuosity per 10 mm Hg increase (95% CI: −0.006 to −0.0006).

Retinal vessel geometry was significantly and independently associated with the SDQ problem score (Table 2). The score increased with 1.1 points (95% CI: 0.3 to 1.9) per IQR increment in fractal dimension and decreased by 1.4 points (95% CI: 2.4 to 0.4) and 0.99 points (95% CI: −0.13 to 2.1) per IQR increment in vascular lacunarity and tortuosity, respectively. Sensitivity analyses showed that these results were driven by the hyperactivity/inattention and conduct problem scales of de SDQ. We found no associations between the retinal width metrics (CRAE or CRVE) and the SDQ problem score (Supplementary Table 1).

**Table 2:** Estimated change (95% confidence interval) overall and subscale problem scores of the Strengths and Difficulties Questionnaire (SDQ) with an interquartile range (IQR) increase in fractal dimension (IQR = 0.04), lacunarity (IQR = 0.06), and tortuosity (IQR = 0.02) of the retinal microvasculature.

**SDQ problem score**	**Retinal microvascular geometry**					
	**Fractal dimension**		**Lacunarity**		**Tortuosity**	
	**Estimated change**	***P*-value**	**Estimated change**	***P*-value**	**Estimated change**	***P*-value**
**Overall problem score**	1.12 (0.30 to 1.94)	0.007	−1.38 (−2.36 to −0.40)	0.006	−0.99 (−2.11 to 0.13)	0.08
**Subscale problem scores**						
Emotional symptoms	0.06 (−0.31 to 0.44)	0.74	−0.13 (−0.58 to 0.32)	0.58	−0.10 (−0.60 to 0.41)	0.70
Conduct problems	0.34 (0.15 to 0.52)	0.0003	−0.23 (−0.45 to −0.003)	0.047	−0.38 (−0.62 to −0.13)	0.003
Hyperactivity/inattention	0.63 (0.21 to 1.06)	0.003	−0.88 (−1.39 to −0.38)	0.0007	−0.53 (−1.11 to 0.05)	0.07
Peer relationship problems	0.09 (−0.15 to 0.32)	0.46	−0.14 (−0.42 to 0.14)	0.33	0.02 (−0.30 to 0.33)	0.92

*Analyses adjusted for sex, age, categorized age-adjusted BMI, maternal occupation, passive smoking, and mean arterial pressure*.

Additionally, retinal vessel geometry was significantly associated with sustained attention, as measured by the Continuous Performance test (Table 3). Mean reaction time on this test increased by 11 ms (95% CI: 4.4 to 17.6) with an IQR increase in fractal dimension. Increases in lacunarity and tortuosity were associated with a decrease in reaction time of 11.1 ms (95% CI: −19.0 to −3.2) and 13.4 ms (95% CI: −21.9 to −4.8), respectively. No significant associations were found between retinal vessel geometry and selective attention measured by the Stroop test or visuospatial analytic ability in the Pattern Comparison test. Increases in fractal dimension tended to decrease visual processing speed as measured by the Digit-Symbol Substitution test, showing an increase in latency by 2.6 s (95% CI: −0.17 to 5.4). An increased tortuosity on the other hand decreased latency by 3.1 s (95% CI: −0.56 to 6.7). Lacunarity was not associated with latency in the digit-symbol substitution test. CRAE or CRVE were not significantly associated with the outcomes of the cognitive performance tests (Supplementary Table 1).

**Table 3:** Estimated change (95% confidence interval) in cognitive performance tests associated with an interquartile range (IQR) increase in fractal dimension (IQR = 0.04), lacunarity (IQR = 0.06), and tortuosity (IQR = 0.02) of the retinal microvasculature.

**Cognitive performance test**	**Retinal microvascular geometry**					
	**Fractal dimension**		**Lacunarity**		**Tortuosity**	
	**Estimated change**	***P*-value**	**Estimated change**	***P*-value**	**Estimated change**	***P*-value**
Stroop (ms)	6.86 (−42.3 to 56.1)	0.78	−14.7 (−73.0 to 43.7)	0.62	−51.6 (−114.7 to 11.4)	0.11
Continuous Performance (ms)	11.0 (4.4 to 17.6)	0.001	−11.1 (−19.0 to −3.2)	0.006	−13.4 (−21.9 to −4.8)	0.002
Digit-Symbol substitution (s)	2.63 (−0.17 to 5.43)	0.07	−2.24 (−5.57 to 1.10)	0.19	−3.06 (−6.67 to −0.56	0.10
Pattern Comparison (s)	0.03 (−0.11 to 0.17)	0.69	−0.02 (−0.18 to 0.15)	0.82	−0.10 (−0.29 to 0.08)	0.26

*Analyses adjusted for sex, age, categorized age-adjusted BMI, maternal occupation, passive smoking, and mean arterial pressure*.

## Discussion

Children aged 8–12 are more likely to have higher problem scores, especially in the hyperactivity/inattention and conduct problem scales of the Strength and Difficulties Questionnaire when they have a denser retinal vascular network. The finding of our study is further supported by the fact that a higher fractal dimension is significantly associated with lower sustained attention, as measured with the Continuous Performance test. The observations are independent of age, sex, age-adjusted body mass index, maternal occupation, passive smoking, and mean arterial pressure.

Consistent changes in the microvascular geometrical pattern were observed in association with behavioral problem scores and cognitive performance outcomes related to attention. A higher fractal dimension, indicating more vessel network complexity, showed a negative association with attention (longer time on Continuous Performance and Digit-Symbol Substitution tests). In a recent study, it was found that patients with major psychoses such as schizophrenia and bipolar disorder have higher retinal fractal dimensions compared to apparently healthy counterparts. Individuals with these affective disorders have symptoms across the attention and behavior spectrum that are often accompanied by vascular co-morbidities. The results suggest that some of these effects can be captured by retinal vessel complexity metrics (38). In contrast, a sparser retinal microvascular network was associated with poorer cognitive performance in older populations (15, 39). Likewise, cognitive dysfunction in about 1,200 participants of the Singapore Malay Eye Study paralleled rarefaction of retinal vessels (13). Then again, McGrory et al. concluded from a study with 700 participants of the Lothian Birth Cohort that quantitative retinal parameters are not significantly associated with cognitive functioning or cognitive change at 70 years of age (40). To the best of our knowledge, we are the first to have investigated an association between retinal vessel complexity and behavior and attention in school children.

Additionally, we found that tortuosity, a proxy for the tortuous character of retinal blood vessels, was inversely correlated with the results on the conduct problem subscale of the SDQ and the outcome of the Continuous Performance test. Along similar lines, Grönland et al. showed a decreased tortuosity of retinal arteries in children (mean age 12 years) with ADHD compared to an age- and sex-matched reference group (20). In a study with adults having bipolar disorder or schizophrenia, a higher retinal arteriolar tortuosity index was observed (41).

Retinal vessel width changes occur in neurodegenerative and behavioral diseases in adults. Smaller venular diameters have been found when comparing patients with Alzheimer to cognitive normal elderly (1). Cheung et al. reported smaller arteriolar diameters and wider venules in Alzheimer patients (14). Wider venules are also suggested to be a marker to psychosis symptoms and anxiety (22, 42). The number of studies investigating retinal microvascular parameters in association with neurobehavioral outcomes at a young age are limited. One study reports an association between wider retinal venular diameters and worse neuropsychological functioning in a population-based cohort of adults approaching midlife (21). Their childhood IQ measured 25 years before was inversely associated with venular diameter. Wei and colleagues report an association between cognitive performance and retinal arteriolar diameter in 11-year old children born preterm and at term (23). Van Aart et al. showed an association between retinal vessel diameters and psychosocial stress in childhood but found no association with SDQ problem scores (43). Similarly, we also found no significant association between retinal vessel diameters and SDQ problem scores or cognitive performance.

The retina is an outgrowth of the embryonic diencephalon and has anatomic similarities and functional characteristics with the brain. Given the fact that retinal vessels resemble their cranial counterparts both in size and functioning, they provide a view on variations in microvascular networks that can be associated with cognitive dysfunction (4). Our results suggest that changes in retinal vascular features may reflect physiological adaptations that are linked to deviations from neurodevelopmental normative trajectories. Unfortunately, the underlying mechanism remains to be elucidated. In support of our reasoning, animal studies confirm that chronic hypoxia during fetal development induces a compensatory increase in brain blood vessel outgrowth (44). Furthermore, an increased vascular complexity was observed in the neonatal rat brain following fetal/neonatal iron deficiency with anemia, (45) which can be associated with brain development deficits and poor cognitive outcomes (46). If our results get further confirmation, then they contribute to the Developmental Origin of Health and Disease hypothesis and provide support for a better understanding of the link between attention disorders and underlying microvascular alterations that affecting oxygen and nutrition supply to the brain.

Our study must be interpreted within the context of its strengths and limitations. The repeated measures of the retinal phenotypes showed a stable geometric pattern of the retinal microvascular network over the time period of a school year, with intraclass correlation coefficients ranging from 0.72 to 0.88. In order to reduce technical variation, the measurements were averaged over the different examinations. Digital fundus photography visualizes a significant part of the retinal microcirculation but because of limitations in image resolution and contrast limits, vessels smaller than 20 μm are usually impossible to identify in a reliable manner. Newer techniques such as optical coherence tomography can bring a solution to also image these tiny blood vessels. However, optical coherence tomography is more challenging to perform on young children during a field study.

The cross-sectional nature of the study is a limitation for drawing conclusions regarding the temporality of the associations. Our participation rate was just under 50%, which limits the representativeness for the population. Low maternal occupation was 17% in our study population while we expected 26% (47). Although we corrected for different correlates, we cannot exclude residual confounding due to unmeasured elements. The children in our study were free of vascular diseases and other major traditional risk factors, hence introducing less confounding (48).

In conclusion, we showed that a denser retinal microvascular network in primary school children is linked to higher score on the SDQ behavioral screening scale and poorer performance on a computer-based test for assessing sustained attention in primary school children. The retinal microvasculature is a proxy for microvascular properties of the brain, and the consequent behavioral and cognitive traits observed here warrant further research into the potential value of an aberrant retinal microvascular network as a phenotype for neurodevelopmental disorders.

## Data Availability Statement

The raw data supporting the conclusions of this article will be made available by the authors, without undue reservation.

## Ethics Statement

The studies involving human participants were reviewed and approved by the Ethics Committees of Hasselt University and Ziekenhuis Oost-Limburg. Written informed consent to participate in this study was provided by the participants' legal guardian/next of kin.

## Author Contributions

EP and NS conducted the field work of the COGNAC study and collected all data. EP and AS analyzed retinal images. EP, PDB, and TN interpreted the results. EP performed statistical analysis and wrote the first version of the manuscript, which was completed by PDB and TN. LI, AS, and NS provided input during the writing process. All authors approved the manuscript for submission.

## Conflict of Interest

The authors declare that the research was conducted in the absence of any commercial or financial relationships that could be construed as a potential conflict of interest.
