# Supplementary material for: Denser Retinal Microvascular Network Is Inversely Associated With Behavioral Outcomes and Sustained Attention in Children
Source: Front Neurol. 2021 Jan 29;12:547033. doi: 10.3389/fneur.2021.547033 (PMC7880124; doi:10.3389/fneur.2021.547033)
Supplement: Supplementary file 1 [file Table_1.docx]

**Table S.1.** Estimated change (95% Confidence Interval) in overall Strengths and Difficulties Questionnaire (SDQ) problem score and cognitive performance tests associated with an interquartile range (IQR) increase in Central Retinal Arterial Equivalent (CRAE) and Central Retinal Venular Equivalent (CRVE). Interquartile ranges of CRAE and CRVE were 17.0 µm and 22.3 µm, respectively. Analyses adjusted for sex, age, categorized age-adjusted BMI, maternal occupation, passive smoking, and mean arterial pressure.

| **Behavior/cognitive performance** | **Retinal microvascular diameters** | | | |
| --- | --- | --- | --- | --- |
|  | **CRAE** | | **CRVE** | |
|  | Estimated change | p-value | Estimated change | p-value |
| **SDQ overall problem score** | 0.65 (-0.38 to 1.68) | 0.21 | 0.72 (-0.23 to 1.67) | 0.14 |
|  |  |  |  |  |
| **Stroop test (msec)** | -14.8 (-71.5 to 41.9) | 0.61 | 29.1 (-25.7 to 84.0) | 0.30 |
| **Continuous Performance test (msec)** | 4.81 (-2.90 to 12.5) | 0.22 | 0.68 (-6.84 to 8.20) | 0.86 |
| **Digit-Symbol Substitution test (s)** | 1.01 (-2.23 to 4.25) | 0.54 | 0.64 (-2.51 to 3.79) | 0.69 |
| **Pattern Comparison test (s)** | 0.067 (-0.095 to 0.23) | 0.41 | 0.021 (-0.14 to 0.18) | 0.80 |
